# Editorial: Positive Technology: Designing E-experiences for Positive Change

**DOI:** 10.3389/fpsyg.2019.01571

**Published:** 2019-07-09

**Authors:** Andrea Gaggioli, Daniela Villani, Silvia Serino, Rosa Banos, Cristina Botella

**Affiliations:** ^1^Department of Psychology, Università Cattolica del Sacro Cuore, Milan, Italy; ^2^Applied Technology for Neuro-Psychology Lab, I.R.C.C.S. Istituto Auxologico Italiano, Milan, Italy; ^3^MySpace Lab, Department of Clinical Neurosciences University Hospital Lausanne (CHUV), Lausanne, Switzerland; ^4^University of Valencia, Valencia, Spain; ^5^CIBER Fisiopatología Obesidad y Nutrición (CIBERObn), Instituto Salud Carlos III, Madrid, Spain; ^6^Universitat Jaume I, Valencia, Spain

**Keywords:** positive psychology, positive technology, user experience (UX), human-computer interaction, positive emotions

While there is little doubt that our lives are becoming increasingly digital, whether this change is for the better or for the worse is far from being settled. Rather, over the past years concerns about the personal and social impacts of technologies have been growing, fueled by dystopian Orwellian scenarios that almost on daily basis are generously dispensed by major Western media outlets. According to a recent poll involving some 1,150 experts, 47% of respondents predict that individuals' well-being will be more helped than harmed by digital life in the next decade, while 32% say people's well-being will be more harmed than helped. Only 21% of those surveyed indicated that the impact of technologies on people well-being will be negligible compared to now (Pew Research Center, 2018).

Although many scientific efforts have been devoted to acknowledging the risks of digital technologies, the question of how computers could be used to improve people's well-being has been much less explored. This was the main motivation for the development of a novel research area—Positive Technology—which aims at investigating how ICT-based applications and services can be used to foster positive growth of individuals, groups and institutions (Botella et al., 2012; Riva et al., 2012; Gaggioli et al., 2017). This area resulted from the convergence of two main trends. First, the emerging interest in the scientific understanding of conditions and processes that contribute to people happiness and well-being, chiefly represented by the fast-growing movement of Positive Psychology. The second trend was the increasing recognition, in the field of Human-Computer Interaction, of the central importance that human experience, values, and ethical concerns have in the design, development and use of interactive systems. The integration of these two perspectives has led to new questions and possibilities concerning how digital technologies could help shaping positive human functioning, strengths, personal empowerment at the individual level, and of groups and organizations, from a social/interpersonal point of view (Botella et al., 2012).

In the last 10 years, research in Positive Technology has attracted increasing attention from an interdisciplinary community of scholars, leading to many conference papers, dedicated symposia and workshops, special issues in journals, and edited books. As an emerging area of research, considerable efforts have been spent on developing conceptual pillars and levels of analysis (Villani et al., 2016; Gaggioli et al., 2017), as well as on the definition of frameworks for bringing well-being principles into the design of interactive systems (Calvo and Peters, 2014; Fleming et al., 2016).

At the methodological and applied level, research on Positive Technology has focused on the design, development, and validation of novel digital experiences that aims at promoting positive change through pleasure, flow, meaning, competence, and positive relationships. These two main facets—theoretical and methodological—of Positive Technology are well reflected by the papers published in this Research Topic, which this editorial aims to briefly summarize.

## Conceptual Frameworks for Using Interactive Technologies for Positive Change

Yaden et al. review the technologies that mental health professionals may use to intervene on and measure well-being, such as predictive algorithms, brain stimulation and virtual reality, and discuss the potential promise and pitfalls of these technologies. While the range of tools that can be used to empower well-being is broad and continues to extend, the authors suggest that safety, effectiveness, and ethical aims are core criteria to be met for the introduction of any technology in people's lives.

Kitson et al. address the important question of how immersive technologies—including virtual, augmented, and mixed realities—can mediate for positive change in users, and which design elements and interaction strategies are best suited to support this process.From this review, these authors provide a set of prescriptive design considerations to serve as tools for designers and developers interested in creating immersive interactive systems and experiences, with the goal of eliciting positive states and supporting positive change.

Filling the gap between existing well-being theories and immediately actionable design practices is a key challenge in Positive Technology. To address this issue, Peters et al. provide a psychological framework grounded in Self-determination Theory (Ryan and Deci, 2017) called Motivation, Engagement & Thriving in User Experience (METUX), which allows HCI researchers and practitioners to design technologies that support psychological well-being and human potential. The model holds that in order to address well-being, psychological needs must be considered within five different spheres of analysis: at the point of technology adoption, during interaction with the interface, as a result of engagement with technology-specific tasks, as part of the technology-supported behavior, and as part of an individual's life overall. These five spheres of experience sit within a sixth, society, which encompasses both direct and collateral effects of technology use as well as non-user experiences.

Diefenbach addresses the question of how psychological knowledge can be translated into technology design, creating synergies between various disciplines. According to Diefenbach, in order to examine this issue, it is important to consider the “bitter-sweet ambivalence of change,” including potential relapses and risks of self-threat, so that technology-mediated interventions adapted from (positive) psychology can have a positive impact to full effect.

## Positive Technology Applications in Mental Health

Positive Technology aims at designing and validating digital well-being interventions that can be effectively used in the prevention and treatment of mental disorders. Enrique et al. describe a pilot randomized clinical trial that evaluated a positive technology application in patients with eating disorders. The experimental application integrated the “Best Possible Self” exercise, a positive psychology intervention that consists in writing and envisioning a future where everything has turned out in the best possible way. The trial involved 54 outpatients who were receiving ongoing specialized treatment in eating disorder services, randomly allocated to either the BPS intervention or to a control condition. Findings showed that both conditions improved over time, but no statistically significant difference was found between the BPS, and control groups.

Villani et al. report results of a controlled study, which tested the efficacy of a 2-weeks e-health Stress Inoculation Training (SIT) intervention on emotion regulation and cancer-related well-being, by comparing it with a control group without such intervention. The experiment involved 29 women with a diagnosis of breast cancer, who had received radical surgery, and who were suitable candidates for adjuvant chemotherapy. Findings showed that patients in the experimental condition did not achieve significant changes related to emotion regulation strategies, but they significantly reduced emotional suppression by 3 months after the end of the intervention. Furthermore, patients in the e-health SIT condition reported a good level of acceptance of the intervention.

Mira et al. describe results of a secondary analysis derived from a randomized controlled trial that tested the efficacy of an internet-based positive psychology intervention designed for patients with depressive symptoms. The intervention consisted of an 8-module Internet-based program, which combined 4 modules based on cognitive-behavioral strategies and 4 modules based on positive-psychology strategies. The clinical study involved 108 patients having minimal, mild, or moderate depressive symptoms. Results showed that patients' negative affect and anxiety decreased significantly during the implementation of the cognitive-behavioral therapy and positive psychology modules. However, depression and positive affect improved only after the introduction of the positive psychology modules.

An interesting development of the use of technology for promoting mental health concerns so-called “serious games,” which are games designed to teach knowledge, skills or behavior change. A key challenge in this domain is how to effectively promote therapeutic games to ensure wide adoption and scalability of this strategy. Poppelaars et al. carried out a study comparing two alternatives in promoting mental health games, one including explicit mental health messaging, and the other not mentioning mental health but highlighting the entertainment value. The experiment involved 129 young adults with mild to mental health symptoms. Participants were shown two distinct trailer designs, but they were unaware that both trailers promoted the same commercial video game. Results showed that young adults with mild to severe mental health symptoms were almost four times more likely to select a game when it was explicitly promoted as beneficial for mental health, compared to when it was promoted as entertaining. Brivio et al. review the concept of technostress—that is, the psychological distress associated with the inability to cope with the use of new technologies—and discuss how Positive Technologies could help preventing this issue, by promoting positive work experiences through an effective organizational safety culture.

Emotion regulation—a person's ability to effectively manage and respond to an emotional experience—is a key aspect of psycho-social functioning and well-being. How could digital tools be used to support this process? Colombo et al. address this question in a perspective article, which discusses the potential of integrating technologies such as virtual reality, wearable biosensors, smartphones, and biofeedback for improving understanding, assessment, and intervention of emotion regulation.

## Positive Technologies for Cognitive Enhancement and Neurorehabilitation

While abundant literature exists regarding the negative psychological implications of videogames, less attention has been dedicated to the potential positive impacts of these technologies on cognitive processes. Milani et al. report results of an experimental study, which investigated the effects of the videogame Tetris in the visuospatial domain both for adolescents and preadolescents, comparing two visualization styles (i.e., 2D vs. 3D). Results showed that playing the Tetris videogame had a positive effect on visuospatial skills—at least in the short term—and that the two visualization formats had a differential influence on these competences.

Interactive technologies such as virtual and augmented reality are increasingly being integrated in treatments aimed at restoring cognitive and motor functions following a neurological damage. In this area, Positive Technology can offer several contributions, e.g., by inspiring the design of ICT-based rehabilitation strategies that support patient's empowerment, engagement and motivation. Perez-Marcos et al. identify four key aspects to consider when designing long-lasting effective treatments for neurorehabilitation: (i) motor-cognitive training; (ii) evidence-based neuroscience principles, in particular those related to body perception; (iii) motivational games; and (iv) empowerment techniques. According to these authors, virtual reality is an effective tool to deliver neurorehabilitation programs because it offers the opportunity to integrate these four assets into a unique training environment. In a similar vein, La Corte et al. describe the significant potential offered by virtual reality to develop applications for the assessment and training of episodic memory in normal and pathological aging, thanks to the possibility of creating personalized and adaptive virtual environments that can simulate naturalistic situations and contexts.

## Positive Technology for Fostering Empathy and Prosocial Behaviors

In the last few years, scientific interest toward the use of advanced simulation technologies, such as virtual, augmented and mixed reality, for promoting prosocial abilities, and behaviors has been increasing. Schoeller et al. discuss the potential of virtual reality, biofeedback, and brain-control interfaces to foster empathic abilities in humans. In particular, they suggest that virtual reality can empower empathy training by allowing users to “embody another self”—i.e., by providing access to sensorial data concerning the body of another person, its immediate context, and peri-personal space. Halton and Cartwright report results of a study, which tested the efficacy of an immersive training intervention to replicate the experience of living with a chronic condition (Inflammatory Bowel Disease). The training intervention, called “In Their Shoes,” draws on the biopsychosocial model of illness and consists of constructed narrative that contains individual challenges typically faced by someone living with Inflammatory Bowel Disease. The digital simulation was delivered via a smartphone application. The study involved 155 employees of a pharmaceutical company and consisted in a pre-post intervention assessment without control condition. Findings showed that the immersive training program led to increased understanding of and empathy for the lived experience of patients with Inflammatory Bowel Disease.

Recupero et al. discuss how mixed reality could be used in combination with storytelling to design experiences that promote cross-cultural integration of immigrants. The authors focus on homesickness (i.e., a state of distress associated to being located in a new and unfamiliar environment) and need of cultural integration as two key dimensions of immigrants' experience. They argue that mixed reality may help addressing these challenges by providing new digitally-augmented tools to improve immigrants' intercultural communication with people in the receiving culture. For example, the use of mixed reality could offer new ways of disclosing cultural meanings, practices, memories, and personal representations of the hosting community, reducing immigrants' feeling of distance and isolation from their countries of origin, and integrating digital storytelling as a practice to make meaning and share experiences of places, events, and people of the new culture.

Żuromski et al. investigate the potential of virtual reality technology to foster empathetic, altruistic, and understanding abilities. In particular, they describe embodied experiences and virtual reality technologies as a form of socially-extended mind and in turn as “mental institutions” that may be used as a model to further our understanding of social phenomena.

## Positive Technology for Promoting Self-Transcendence

A recent development of Positive Technology concerns how interactive technologies may be used to promote self-transcendent emotional experiences, that is, out-of-the-ordinary life moments that allow individuals experiencing something greater of themselves, reflecting on deeper dimensions of their existence, shape lasting spiritual beliefs, and enhance feelings of connectedness (Gaggioli, 2016; Kitson et al., 2019).

Two papers investigated how virtual reality can be used to promote and assess awe, a complex emotion with a significant transformative potential. Chirico et al. describe results of a study, which tested the efficacy of virtual environments designed to elicit awe experiences. In this experiment, which involved 36 participants, three virtual environments were designed to induce awe, whereas the fourth was targeted as an emotionally-neutral stimulus. Results showed that virtual environments designed for enhancing perception of vastness and need for accommodation—two key dimensions of awe—induced higher awe and sense of presence compared to the neutral virtual environment. Quesnel and Riecke investigated how virtual reality may elicit awe, and how features of aesthetic beauty/scale, familiarity, and personalization (i.e., self-selection of travel destinations) may elicit this complex emotion. In their study, 16 participants were presented with a virtual environment that allows for the appraisal of the Earth's landscapes, cityscapes, and a view of the planet from Earth's orbit. To test participants' awe intensity, the authors used frequency of goose bumps for each participant using a custom-made “goosecam” as well as self-report questionnaires to collect participants' emotion ratings of the virtual experience; in addition to these quantitative measures, qualitative methods of open-ended interviews and observations of the participants were used. Results of the experiment showed that immersive virtual reality can elicit subjective experiences of awe and physiological goose bumps were observed in several participants. Furthermore, findings revealed that aesthetic beauty/scale and familiarity/personalization of the environment positively influenced awe.

Stepanova et al. provide a design framework to create virtual reality experiences of the “Overview Effect,” a profound cognitive shift reported by many space-travelers triggered by the sight of the earth from beyond its atmosphere. Common outcomes of the experience are a feeling of awe for the planet, an enhanced feeling of interconnectedness and a renewed sense of responsibility for taking care of the environment. After reviewing key psychological studies on the Overview Effect and awe, authors propose guidelines for creating virtual reality experiences to elicit this experience and evaluation methods for assessing it.

## Conclusions

The papers published on this Research Topic provide a broad overview of Positive Technology and indicate directions for its future developments. The diversity of frameworks, applications areas, intervention protocols and technological solutions covered by these studies suggest that this emerging field offers exciting new avenues for research and innovation in the domain of digital well-being. Furthermore, the analysis of these contributions allows identifying some key challenges for the future evolution of this area.

First, the variety of conceptual analyses published in this Research Topic confirms the inherently interdisciplinary nature of Positive Technology, emphasizing the plurality of perspectives (i.e., psychological, neuroscientific, technological, design, artistic) that play a role in the design of digital well-being tools. On the one side, the richness of these diverse views offers the unique opportunity to create new synergies between different research communities, with the common goal of improving the relationship between people and technology and human functioning in general.

On the other, renewed efforts are required to create common theoretical and methodological approaches, also in terms of developing interdisciplinary education programs to train core skills and competences to psychologists, designers, and practitioners interested in this topic. Furthermore, as emphasized by Peters et al. achieving a stronger connection between frameworks and actionable design practices is a key research objective.

A further important trend highlighted by the contributions included in this Research Topic is that applications of Positive Technology are growing and wide-ranging, encompassing mental health, neurorehabilitation/cognitive enhancement, multicultural integration, socio-cognitive skills, and education. However, several of these applications are still conceptual and little or no empirical work exists on benefits, thus an important future challenge is to translate these scenarios into testable tools, protocols, and services.

A third and final consideration that can be drawn from the present Research Topic is that more emphasis should be placed on experiences, rather than on technologies, when designing for positive change. In this regard, the definition “Positive Technology” could be misleading, as a technology is neither positive nor negative: its valence is ultimately determined by the experience that a person has when interacting with a digital tool or content, to which extent this experience is meaningful and relevant and can contribute to a positive change for that person. Thus, a future goal for Positive Technology researchers is to better understand which dimensions of digital tools can promote positive change through pleasure, effort, challenges, flow, meaning, competence, endeavor, and positive relationships.

In conclusion, this Research Topic provides an overview about the state of the art of research in technology for mental well-being and offers possible directions to guide the design of future applications and services in this area. Although these contributions have highlighted several tools—such as virtual/augmented reality, biosensors, smartphones, videogames—which can be used for promoting positive change, the range of technologies that can be used for this purpose is steadily growing and potentially extends to robotics, artificial intelligence, and neurotechnologies. In this perspective, we believe that Positive Technology represents not only a scientific, but also a cultural opportunity to promote a more human-centered view on the development of our digital future.

## Author Contributions

All authors listed have made a substantial, direct and intellectual contribution to the work, and approved it for publication.

### Conflict of Interest Statement

The authors declare that the research was conducted in the absence of any commercial or financial relationships that could be construed as a potential conflict of interest.
